# Response to commentaries on our review of Fast Mapping in adults

**DOI:** 10.1080/17588928.2019.1651709

**Published:** 2019-08-09

**Authors:** Elisa Cooper, Andrea Greve, Richard N. Henson

**Affiliations:** a Medical Research Council Cognition and Brain Sciences Unit, University of Cambridge, Cambridge, England; b Department of Psychiatry, University of Cambridge, Cambridge, England

**Keywords:** Fast Mapping, Episodic Encoding, Hippocampus, learning, memory

## Abstract

We thank all the commentators for their thoughts on our review of Fast Mapping (FM) in adults, where we questioned the evidence that FM is a distinct learning mechanism, and urged caution over the excitement generated by the original report of FM in adults with amnesia using the fast mapping paradigm (FMP) . While some commentators remain convinced that there is good evidence to support a FM process in adults, most reported a skepticism similar to ours. Here we respond to the main comments, and clarify some of the terms of debate.

## Terminology

As is common in such debates, it is vital to define the terminology. We agree with Gernsbacher and Morson (this issue) that fast mapping is a term whose meaning has been confounded and extended. We were guilty in our review of conflating the concept of FM (as a learning process distinct from conventional episodic encoding or word acquisition) and the specific paradigm used to engender FM that was introduced by Sharon, Moscovitch, and Gilboa (2011). For clarity, we will use different abbreviations for each: FM for the theoretical fast mapping process (FM), and FMP for the fast mapping paradigm. In fact, apart from briefly considering the developmental literature, all the evidence we reviewed in adults was restricted to variants of Sharon et al.’s FMP. Thus, while we remain skeptical that their FMP engenders distinct types of learning, we accept that it is possible that FM could occur in other paradigms and situations, as Warren and Duff (this issue) and Koutstaal (this issue) politely point out. Indeed, fast mapping may require the embodied, social factors considered by Koutstaal; a possibility that definitely warrants further investigation (though see commentary by O’Connor, Lindsay, Mather, & Riggs, this issue, for arguments against any existence of FM in adults or infants).

Furthermore, when discussing FM, we agree with Warren and Duff (this issue) that it is important to distinguish the simple (possibly episodic) association of an unfamiliar word with an unfamiliar picture, as assessed in Sharon et al.’s FMP, from the long-term lexicalization of a new word (what Warren and Duff call WL_FM_): word-learning may sometimes occur through FM, even if such word-learning, as distinct from episodic association, does not occur in the FMP. Having said this, the implicit memory tests added to the FMP by Coutanche and Thompson-Schill (2014) and Coutanche & Koch (2017)﻿ are often thought to index lexicalization; a point we return to below.^1^1Coutanche (this issue) suggests that above chance performance in the FM condition of the FMP is, on its own, sufficient to demonstrate that FM occurs. However, we are not aware that anyone ever claimed this was a useful definition of FM, because such performance is no different from what has been investigated in hundreds of incidental associative memory or word-learning experiments. Rather, the real question, as Coutanche later argues, is whether FM is a qualitatively different process from normal episodic encoding, or from normal word learning.

Finally, Coutanche (this issue) highlights the importance of separating the neural properties of FM (e.g., independent of hippocampus; dependent on anterior temporal lobe, ATL) from its cognitive properties (e.g., sensitive to interference; insensitive to sleep). We agree and made the same point in the conclusion of our original review. We should also point out that we do not dispute the possibility that hippocampal-independent, cortical learning can occur (e.g., over years in developmental amnesia; Elward, Dziezciol, & Vargha-Khadem, this issue); we only dispute that this occurs as rapidly as originally claimed in the FMP. While Coutanche (this issue) cites the ‘Complementary Learning Systems’ (CLS) model as supporting the possibility that cortex can learn information relatively quickly when that information is consistent with prior knowledge (McClelland, 2013; Kumaran & McClelland, 2016; see also commentary by Mak, this issue), learning in this model still requires many episodes of training (or cortical replay) of interleaved new and old patterns, which would surely require more than the one or two encoding trials and 10 minutes between study and test in the FMP.^2^2While Coutanche (this issue) is reassured by similarities between the factors claimed to affect FM and the factors relevant to the CLS model, Gaskell and Lindsay (this issue), in their response to our review state the opposite, i.e., the basic FM effect is contrary to CLS. While this disagreement probably relates to FM’s cognitive versus neural properties, we place more value on empirically reproducible findings than on consistency with an existing theory, given the reproducibility problem in psychology (Open Science Collaboration, 2015) and given that it is relatively easy to find a similar theory somewhere in the literature (and some of these theories may themselves be based on unreliable results).

## FM in amnesia

We were very interested to read of Elward et al.’s (this issue) new data from three individuals with developmental amnesia (DA), who sustained hippocampal damage in early life (before vocabulary acquisition). That these individuals can learn new words supports the idea of hippocampal-independent learning, even if that learning is not necessarily ‘fast’. However, when learning must be fast, as in the FMP, Elward et al. report that the DA individuals showed the same pattern of explicit memory performance that we described in our review and that has been found in all labs outside the lab that produced the original Sharon et al. (2011) study – viz worse memory performance under the FM condition than the EE condition, in both patients and controls.

Zaiser et al. (this issue) echo Elward et al.’s point that performance in FM conditions might also benefit from extra-hippocampal structures like perirhinal cortex, which is believed to support semantic processing and familiarity signals. The implicit tests of lexical processing introduced by Coutanche and Thompson-Schill (2014) might be affected by such perirhinal processes, though we do not think this can explain the FM advantage reported by Sharon et al. (2011) in explicit tests such as 3AFC, since this requires memory for associations between objects and names, which is conventionally associated with recollection and hippocampal function.

Gilboa (this issue) questions the data from the three adults with acquired amnesia that we reported, arguing that at least one of them (P5) shows a pattern similar to Sharon et al. (2011). We prefer not to make inferences about single-cases when they have only been tested once^3^3We do value single-case studies when the individual has been tested repeatedly, such that measurement noise and systematic confounds have been addressed. since P5’s data are likely to include measurement error, and are not controlled for task order, stimulus set assignment, etc. This is why we put more emphasis on the group pattern (albeit only from 3 cases), where the patient group scored worse than controls on average under both FM and EE conditions. The difference in group means was numerically smaller for FM than EE, and the group-by-condition interaction might be significant with more patients. However, it is not the cross-over interaction reported by Sharon et al., and could easily be explained by a floor effect, given that FM is worse than EE on average across both groups (49% versus 65%, respectively) and chance is at least 33% (see section below about baseline rates).

We look forward to seeing Gilboa’s data from individuals with Mild Cognitive Impairment (MCI), because if they show the same cross-over interaction as Sharon et al. (2011), where the MCI patients show better performance for FM than EE (or at least, a group-by-condition interaction that cannot be simply attributed to a floor effect on the FMP’s FM condition), then this will definitely re-invigorate the theoretical and practical importance of the FMP (particularly since there is a larger population from which to recruit MCI patients relative to patients with acquired hippocampal damage, which could resolve the current statistical problems in the FM-amnesia literature).^4^4It is interesting that Coutanche (this issue) questions the severity of amnesia in some patients with hippocampal damage who have been run on the FMP, like those we described in our review, and suggests that FM could be only observed with more severe cases. Putting aside the tricky question of how to define severity, if he is correct, then one would not expect to see any evidence of FM in MCI patients, whose memory deficits are generally less pronounced that individuals with acquired hippocampal damage.

## Measuring FM using implicit tests of memory

The dissociation between FM and EE conditions in terms of implicit measures, as introduced to the FMP by Coutanche and Thompson-Schill (2014), brought the FM debate back from episodic memory in amnesia to the long tradition of word-learning. As Gaskell and Lindsay (this issue) point out, and as we mention in our original review, the lexical competition effects on reaction times (RTs) of the type Coutanche and Thompson-Schill used as evidence of lexical integration, are normally only found after a period of consolidation, such as overnight sleep.^5^5Gaskell and Lindsay (this issue) acknowledge that competition effects are sometimes found on the same day, but these have been found even under EE conditions, and it is possible that some competition effects do not uniquely index lexical integration. Indeed, Gaskell and Lindsay say they tried to replicate Coutanche and Thompson-Schill’s effects using either written or spoken words but found no evidence of same-day competition in either FM or EE conditions. When using spoken words (more similar to the original fast mapping experiments in children), they say they did find evidence of competition after one night’s sleep (and still after a week), but this was in both FM and EE conditions, which argues against any special status to FM encoding (Walker et al., 2019).

Unlike Gaskell and Lindsay (this issue), Zaiser, Meyer, and Bader (this issue) say they did find same-day lexical competition effects after FM encoding (see https://www.biorxiv.org/content/10.1101/594218v1, Zaiser, Meyer, and Bader, 2019 for a preprint). Unfortunately, in their Experiment 1, they did not include an EE condition, so one cannot conclude that their competition effect was specific to a fast mapping condition. In their Experiment 2, they switched to a (facilitatory) RT measure of semantic priming, defined as whether the category of a prime (new word) matched that of the target (a familiar word). With this measure, they did find a priming effect that was selective to the FM but not EE condition, but only when the FM referent object shared semantic features with the unfamiliar object (e.g., from the same category, like birds). Interestingly, they found this facilitatory priming effect on same-day testing, but not after sleep, which is the opposite pattern to that found by Coutanche and Thompson-Schill (2014) (which Zaiser et al. suggest is because Coutanche and Thompson-Schill did not counterbalance their stimuli).

Taken at face value, Zaiser et al. (this issue)’s results support their neuroscientific claim, and the related computational claim of Mak (this issue), that feature overlap between the unfamiliar and known object in the FMP (which are from the same category) is important for rapid learning. However, this seems to conflict with Coutanche and Koch (2017) finding of lexical competition only when the semantic referent was atypical for its category. While Zaiser et al. and Coutanche (this issue) point out that the typicality of an object (with respect to all objects of the same category) is logically independent of the degree of feature overlap between that object and the particular unfamiliar object used in the FMP, it seems likely that, unless specifically matched, atypical objects would share fewer features with an unfamiliar object of the same category than would typical objects (Rosch & Mervis, 1975).

In our own work, we also failed to replicate the original Coutanche and Thompson-Schill (2014) same-day lexical competition effect, but did find evidence of ‘semantic’ priming as a function of whether or not the category of the (hermit) word, presented for the natural vs man-made decision task, matched that of the unfamiliar natural object with which the hermit’s neighbor was paired during FM encoding. We are currently running a pre-registered online replication and extension of this experiment (https://osf.io/atkp4, Cooper, Greve, and Henson, 2019b) and hope to share the results soon.

In summary, while one should wait for the results from Gaskell and Lindsay (this issue), Zaiser et al. (this issue), and our own work to be peer-reviewed, there seems little doubt that the current pattern of FM results using implicit memory measures is complex, and it remains unclear whether FM and EE dissociate. This confusion is probably because implicit RT measures are noisy, i.e., have high measurement error (Buchner & Wippich, 2000), and so studies with greater statistical power, and/or formal meta-analyses appear necessary.

## Paradigm (FMP) issues

We agree with Zaiser et al. (this issue) that it is likely to be important that learning in the FM condition is incidental, otherwise performance can be contaminated by intentional strategies. The fear that the explicit memorization instructions in the EE condition could encourage such intentional strategies in the FM condition is why many studies administer it before the EE condition. A downside of this however are the potential confounds of task order, e.g., differential pro/retroactive interference, differential practice or fatigue, etc. Thus, future studies (at least those where many participants can be tested, e.g., healthy people or MCI patients) should consider running FM and EE conditions in separate groups of participants (as in Experiment 4 of Cooper et al., 2019a).

Another important issue for future use of the FMP concerns the novel word stimuli. Most studies have used the real names of rare objects. Although those studies often include an additional familiarity test that allows objects that participants did know before the experiment to be excluded from analysis, there are likely to be morphological features of those names (e.g., ‘mango’ in mangosteen) that are more common for one category (e.g., fruit) than another (e.g., animals). If the 3AFC lures are from different categories, then this could cause above chance (33%) performance even for people who never participate in the study phase. Thus, baseline memory performance should be measured, as in Smith, Urgolites, Hopkins, and Squire (2014) who reported baseline rates for the original FMP stimuli of nearly 40%, and memory performance adjusted for such baseline rates. Failing this, the names should at least be counterbalanced across EE and FM conditions (which was not the case in the original Sharon et al., 2011, study), to prevent any artefactual differences between conditions. Alternatively, one could use novel pseudowords for object names, which should not be pre-experimentally familiar to anyone and could be chosen not to share morphological features with object categories.

Finally, we do not dispute the claims of Gilboa (this issue) that sensitivity to interference is an important feature of FM; this dissociation of FM and EE according to interference simply requires replication in other labs (while also controlling for other factors, e.g., task order and stimulus sets). By doing so, boundary conditions can be established that can both explain the divergent results across previous studies and inform future studies.

## Conclusion

In conclusion, we stand by our original claim that the evidence for fast mapping (FM), at least in adults within the fast mapping paradigm (FMP) introduced by Sharon et al. (2011), is not convincing, and we are comforted that most of the commentators seem to agree with this. However, we are scientists who are perfectly happy to change our minds if new evidence comes to light, particularly if that evidence is replicated within and across different laboratories.
